# Advances in Liquid Crystalline Epoxy Resins for High Thermal Conductivity

**DOI:** 10.3390/polym13081302

**Published:** 2021-04-15

**Authors:** Younggi Hong, Munju Goh

**Affiliations:** Department of Chemical Engineering, Konkuk University, 120 Neungdong-ro, Gwangjin-gu, Seoul 05029, Korea; hongyg21@konkuk.ac.kr

**Keywords:** thermal conductivity, epoxy, liquid crystal, composite, epoxy mold compound

## Abstract

Epoxy resin (EP) is one of the most famous thermoset materials. In general, because EP has a three-dimensional random network, it possesses thermal properties similar to those of a typical heat insulator. Recently, there has been substantial interest in controlling the network structure of EP to create new functionalities. Indeed, the modified EP, represented as liquid crystalline epoxy (LCE), is considered promising for producing novel functionalities, which cannot be obtained from conventional EPs, by replacing the random network structure with an oriented one. In this paper, we review the current progress in the field of LCEs and their application to highly thermally conductive composite materials.

## 1. Introduction

Epoxy resin (EP) based thermosetting polymers are used in various industries because of their excellent adhesion, thermal resistance, chemical resistance, and mechanical strength owing to formation of a three-dimensional network structure with curing agents such as acid-free amine phenol [1,2,3,4]. Recently, the demand for materials that efficiently dissipate heat owing to their high thermal conductivities has been increasing, driven by the development of high-power next-generation electronics that are lighter, smaller, and thinner [5]. Therefore, good thermal conductivity is required for EP to be applicable as a high-performance heat-dissipating material.

Heat transfer of cured EP is facilitated by the transfer of phonon oscillations in the crystalline and non-crystalline regions. It is expressed by the *Debye* equation.

(1) $$\lambda =\left(\frac{1}{3}\right)\times C_{v}\times u\times l$$

*Debye* Equation (1) has several variables: l is the mean free pathway of a phonon, u is the sound velocity, $C_{v}$ is the volumetric heat capacity that determines the polymer properties, and λ is the thermal conductivity of the polymer. Although $C_{v}$ is determined by the properties of the resin, l is determined by the crystallinity and orientation of the cured EP [6,7,8,9,10]. To achieve high thermal conductivity, the variable l needs to be increased. This makes it necessary to minimize static scattering in the crystalline region of phonons and dynamic scattering related to orientation.

To this end, we can improve the thermal conductivity of polymers by enhancing the crystallinity of polymeric materials. For example, high thermal conductivity and crystallinity were obtained through polyethylene chain orientation [10], improved π−π stacking of conjugated polymers [11], and by means of hydrogen bonds of non-crystalline polyvinyl alcohol [12].

However, EP lacks crystallinity and molecular orientation due to the formation of a three-dimensional network structure, which results in thermal insulation [13]. In this review, we provide an overview of studies aimed at improving the thermal conductivity of EP by introducing liquid crystallinity into the molecular structure in order to complement the many studies that have attempted to ascertain the thermal properties of EP arising from the three-dimensional network structure. In addition, the molecular design of liquid crystal epoxy, stable liquid crystal temperature expression, curing of liquid crystal epoxy, and analysis of thermal properties are also introduced.

**Figure 1:**
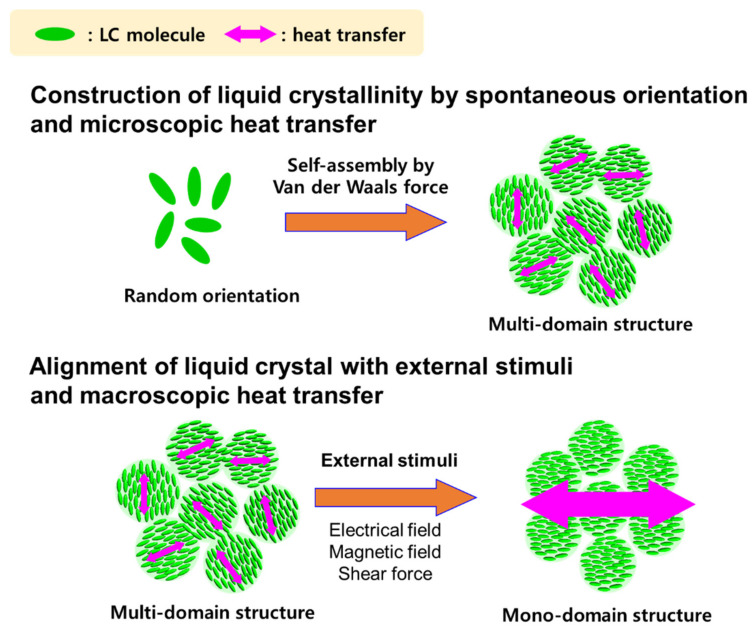
Schematic explanation of correlation between liquid crystal structure and thermal conductivity.

Liquid crystalline epoxy (LCE) molecules have self-assembling capabilities, which impart self-orientation properties and a distinctive orientation in the micro-areas. Therefore, the mean free pathway of a phonon is increased, thus improving the thermal conductivity [14,15,16,17,18,19,20,21,22,23]. Figure 1 shows the basic molecular structure and concept of LCE. The three-dimensional network of the existing EP is a structure that causes a considerable amount of scattering while restricting easy transfer of phonons, which is essential for heat transfer. However, LCE has a molecular structure that can reduce phonon scattering and increase crystallinity in microscopic regions due to the domain structure formed by the self-assembly of liquid crystal molecules. As a result, it is possible to improve the thermal conductivity, and rod-shaped LCE exhibits a thermal conductivity higher than 0.4 W/mK, which is twice as large as the thermal conductivity observed for diglycidyl ether of bisphenol A EP [18].

## 2. Molecular Design of Liquid Crystalline Epoxies

**Figure 2:**
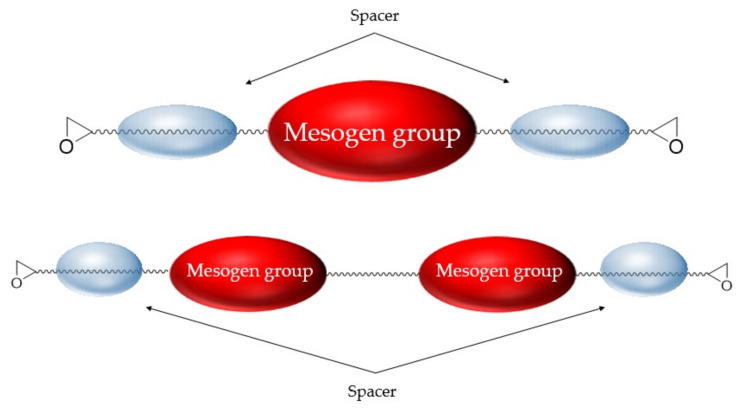
Typical molecular structure of liquid crystalline epoxy resin.

The molecular structure of a typical rod-shaped LCE consists of a mesogenic core represented by a rigid molecular structure controlling assembly between molecules through π−π interaction and a flexible sp^3^ hybridized spacer that prevents crystallization by applying steric hindrance between the assembled molecules. In addition to the basic molecular structure of liquid crystal formation, LCE has a molecular structure that introduces two or more epoxide groups, which are responsible for curing the EP. Figure 2 shows the basic molecular structure of LCEs. Typical LCE resins are rod-shaped, low-molecular-weight monomers that are different from conventional EP molecules. Mesogenic groups and flexible chains exist between epoxy groups [19]. In addition, the twin mesogenic epoxy monomer has a structure in which a flexible chain exists between the mesogenic groups [24]. The properties of LCE tend to depend on the flexibility of the mesogenic group and spacer attached to the epoxy group; therefore, the structure and properties of the mesogenic group are important factors affecting the thermal conductivity of LCE [25,26].

**Figure 3:**
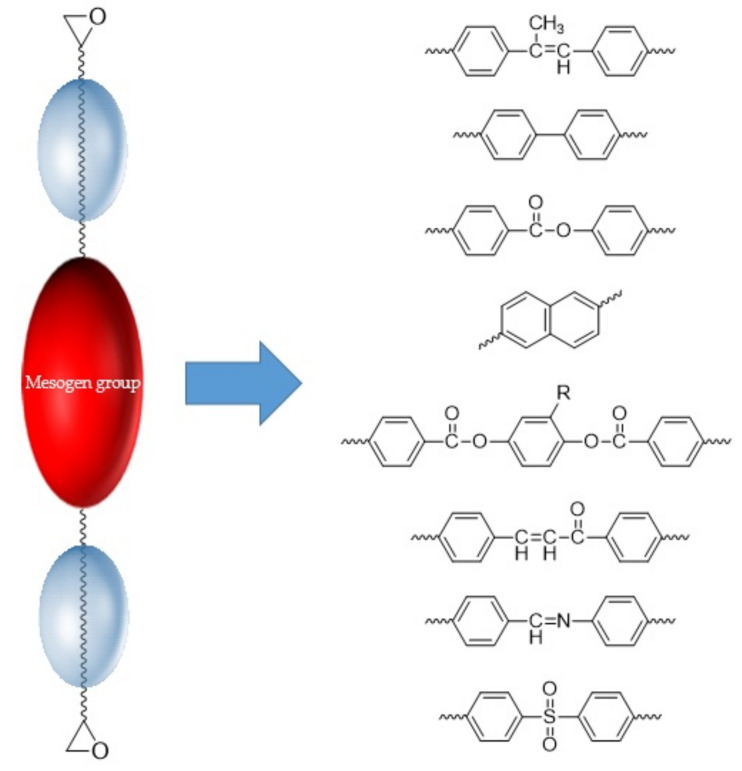
Typical molecular structure of mesogenic groups.

Figure 3 shows a representative LCE mesogenic group. In addition, it is possible to form a three-dimensional network structure through the curing reaction of LCE and amine-based curing agents [26]. The molecular structure of the mesogenic core features a large aromatic ring structure with high planarity to generate strong π–π interactions. It provides the fundamental driving force for self-assembly in LCE and simultaneously has a key role in increasing the length of the mean free pathway of the phonon, which is the most important factor affecting thermal conductivity. As the size of the aromatic group increases, the length of the mean free pathway increases; therefore, several studies have been conducted to increase the number of aromatic rings in the mesogenic core.

**Figure 4:**
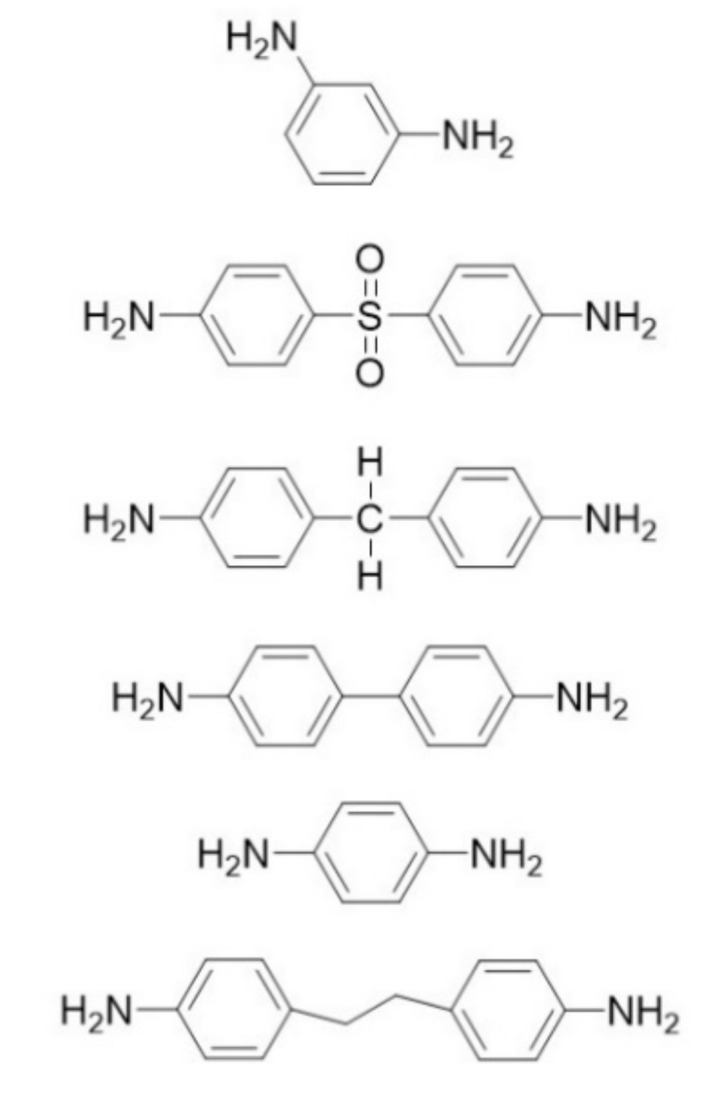
Typical molecular structure of amine-based curing agents.

Figure 4 shows some representative amine-curing agents used. Not all amine-based curing agent react with epoxy groups. However, if a reaction occurs, curing can be observed directly through a polarization microscope, and the mesophase of the liquid crystal can also be observed using a polarization microscope. The structure of the epoxy during curing can be controlled by regulating the temperature at which curing is performed. If the liquid epoxy is cured in the temperature range for liquid crystallinity, the liquid epoxy is cured in the oriented state; therefore, it remains oriented at the end of the curing process.

**Figure 5:**
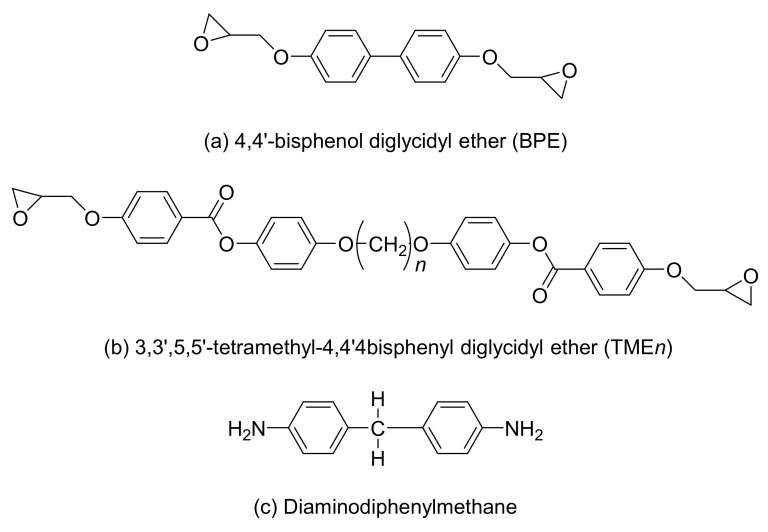
Molecular structures of (**a**) 4,4′-biphenol diglycidyl epoxy (BPE), (**b**) 3,3′,5,5′-tetramethyl-4,4′4bisphenyl diglycidyl ether (TME*n*) (*n* = 4, 6, 8), and (**c**) diaminodiphenylmethane (DDM).

Some studies have shown that two phenyl benzoates are used as the mesogen group; an LCE (TME*n, n* = 4, 6, 8) was synthesized wherein epoxy groups exist at both ends, and the aliphatic chains between the mesogen and epoxy groups were used as spacers [7]. The thermal conductivity of the LCE, which was cured using diaminodiphenylmethane (DDM) as a curing agent, was measured. In addition, 4,4′-biphenol diglycidyl epoxy (BPE) was cured with DDM to compare its thermal conductivity. Figure 5 shows the structures of BPE, 3,3′,5,5′-tetramethyl-4,4′4bisphenyl diglycidyl ether (TME*n*)(*n* = 4, 6, 8), and DDM.

**Figure 6:**
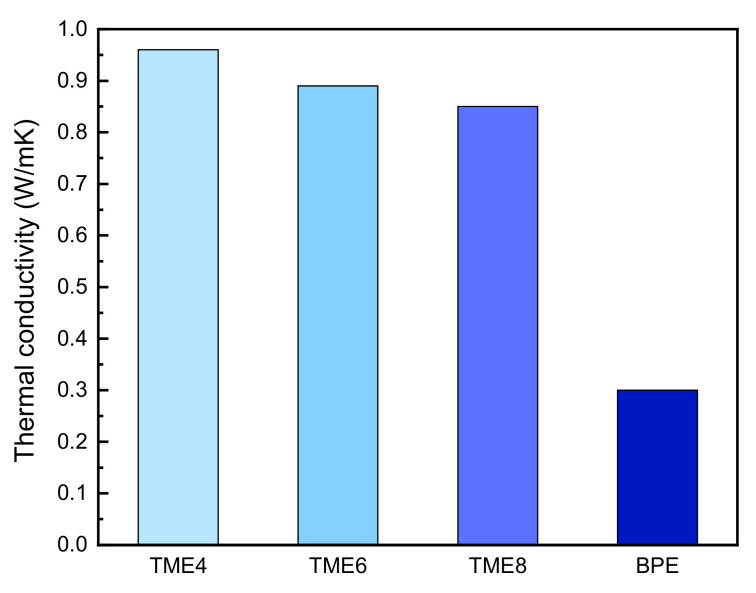
Thermal conductivities of TME*n* (*n* = 4, 6, 8) and BPE.

The epoxy monomer was mixed with a curing agent. The mixture was heated for hours in an oven at 175 °C, and the thermal conductivity was calculated using the heat-diffusion coefficient and heat capacity. The highest thermal conductivity of TME4 is found to be 0.96 W/Mk and approximately three times higher than BPE. TME6 and TME8 also showed higher thermal conductivities compared to BPE. Figure 6 summarizes the thermal conductivities of TME*n* (*n* = 4, 6, 8) and BPE.

Comparing the thermal conductivities of the TME*n* epoxy composites, the length of the spacer between the mesogenic groups is found to have a significant effect on the thermal conductivity. In other words, the closer the distance between the mesogenic groups, the closer the structure becomes to a crystalline one, which leads to a higher thermal conductivity. In TME4, the concentration of the phenyl benzoate group forming the domain was higher than that of the other epoxy, resulting in a higher thermal conductivity. We also confirmed that using LCE resulted in a significantly higher thermal conductivity than using a commonly used commercial EP (0.17 to 0.21 W/mK). When we compared materials mixed with bisphenol A and TME8 with DDM, TME8/DDM was observed to have a lattice structure, and bisphenol A/DDM was found to have an amorphous structure.

This anisotropy also tends to be better when magnetic or electric fields are applied. If such fields are applied to the liquid epoxy during the curing time, the molecules are aligned along the direction of the applied fields. Therefore, orientation of the state is improved under magnetic or electric fields [18,27,28].

## 3. Liquid Crystalline Epoxies Bearing Diglycidyl Moieties at the Side Positions

**Figure 7:**
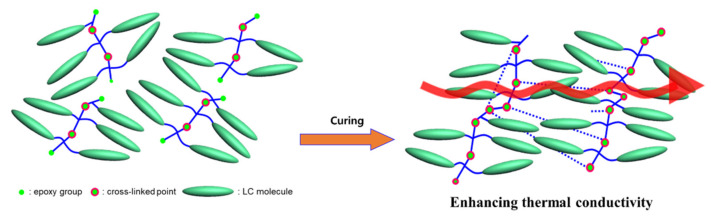
Schematic explanation of the correlation of liquid crystalline epoxy (LCE) bearing diglycidyl moieties at the side positions with thermal conductivity.

LCE resin induces spontaneous molecular orientation through π–π interactions between the mesogenic structures, thereby improving the crystallinity of the resin. Molecular orientation enables efficient heat transfer through phonon vibration; thus, it is a critical factor for determining the thermal conductivity of both amorphous and crystalline matter. For example, the thermal conductivity of the cured LCE with the biphenyl mesogenic group could be improved by 30% compared to that of a non-crystalline EP, which is ascribed to the LC orientation [29]. However, typical LCEs bear epoxies as end groups. Consequently, the LC orientation could be destroyed by curing the LCEs using a typical linear diamine curing agent. Therefore, new molecular designs are required to produce thermally conductive LCEs with a maintained LC orientation upon curing them with existing curing agents. In addition, the exothermic epoxy curing process may generate sufficient heat to perturb the oriented LC phase. To maintain the LC phase through the exothermic curing process, stronger intermolecular attractive forces than π–π interactions between LC molecules are required. To this end, a new series of LCEs possessing epoxies at the molecular side positions and cyanobiphenyl mesogenic end groups with intermolecular dipole–dipole and π–π interactions were designed and synthesized [30,31]. Figure 7 shows a schematic explanation of the correlation of LCE bearing diglycidyl moieties at the side positions with thermal conductivity. Even after the curing reaction, the stable π–π interaction of the liquid crystal group is possible, forming a molecular structure that forms a heat transfer pathway.

**Figure 8:**
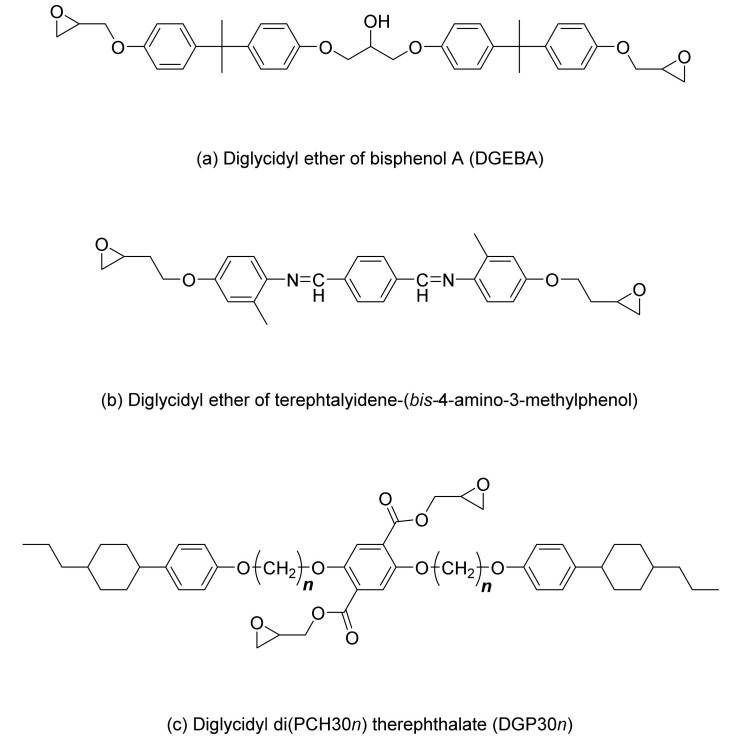
Molecular structure of (**a**) diglycidyl ether of bisphenol A (DGEBA), (**b**) diglycidyl ether of terephthalyidene (DGETAM), and (**c**) diglycidyl diphenylcyclohexyl (PCH30*n*) phthalate (DGP30*n*). Reproduced from Ref. [31] with permission from The Royal Society of Chemistry.

Synthesized diglycidyl diphenylcyclohexyl (PCH30*n*) phthalate (DGP30*n*) represents a new liquid crystal epoxy [31], where PCH30*n* indicates the number of methyl groups attached to 4-(trans-4-*n*-propylcyclohexyl) phenol (PCH300). Figure 8 shows the structures of diglycidyl ether of bisphenol A (DGEBA), diglycidyl ether of terephthalyidene (DGETAM), and DGP30*n*.

**Figure 9:**
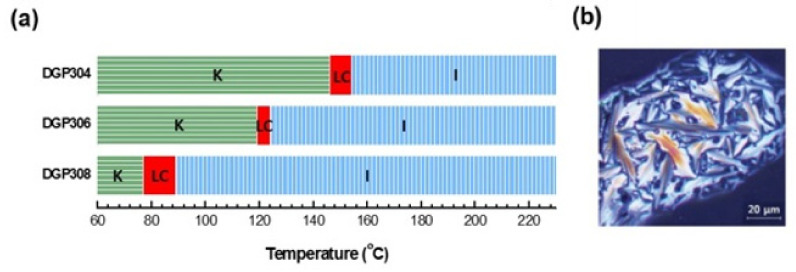
(**a**) The transition temperature of DGP30*n* (*n* = 4, 6, 8) and (**b**) DGP308′s polarization microscopy (POM) images. Reproduced from Ref. [31] with permission from The Royal Society of Chemistry.

The phase transition temperatures of DGP304, DGP306, and DGP308 were determined via DSC measurements. The parameters K, S_x_, and I represent the crystalline, smectic, and isotropic phases, respectively. The enthalpy changes associated with the phase transition temperature are shown in Figure 9. Fan-shaped textures were observed in all polarization microscopy (POM) photographs of DGP30*n*, which show that all DGP30*n* structures appear as highly regularized smectic structures during heating and cooling.

To obtain a high thermal conductivity from LCE, it is necessary to obtain cured EP that maintains the orientation of the liquid crystal. While LCE is being cured, it is necessary to cure it in the liquid crystal section to maintain its orientation [19]. However, existing thermosetting LCEs have the disadvantage that the phase transition temperature range of the liquid crystal is narrow, making it easier to phase in isotropic form on the liquid crystal due to the exothermic curing reaction.

In other words, the available temperature range has always been a problem owing to its narrowness. In this study, each of the synthesized DGP30*n* epoxies exhibited a stable smectic phase; however, the liquid crystal phase existed at a temperature less than 10 °C, i.e., a very narrow temperature range. This narrow temperature range is not suitable for curing EPs because the emitted heat can destroy liquid crystals during curing and convert them to an isotropic form. Therefore, the liquid crystal temperature range of the liquid crystal epoxy should be widened to maintain the liquid crystalline phase during heat curing.

**Figure 10:**
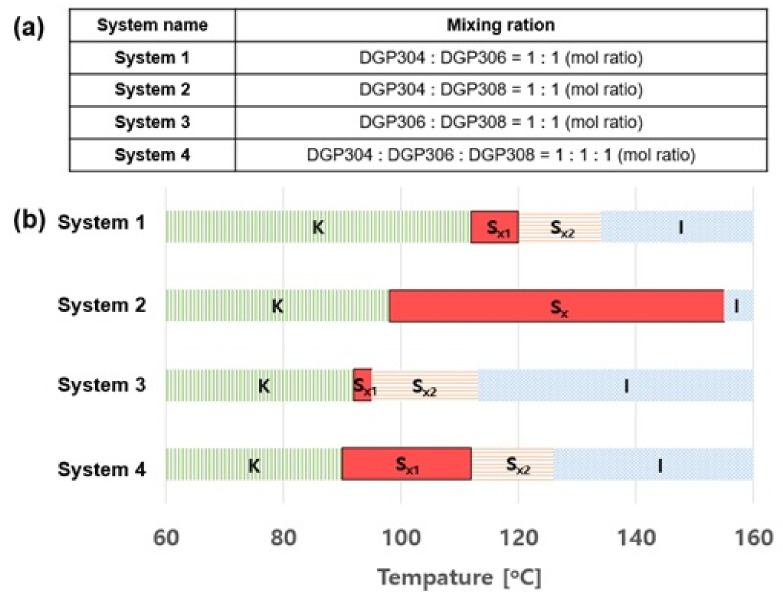
(**a**) Several systems that are mixed in the same ratio as that of DGP30*n*. (**b**) Transition temperatures of these systems. Reproduced from Ref. [31] with permission from The Royal Society of Chemistry.

**Figure 11:**
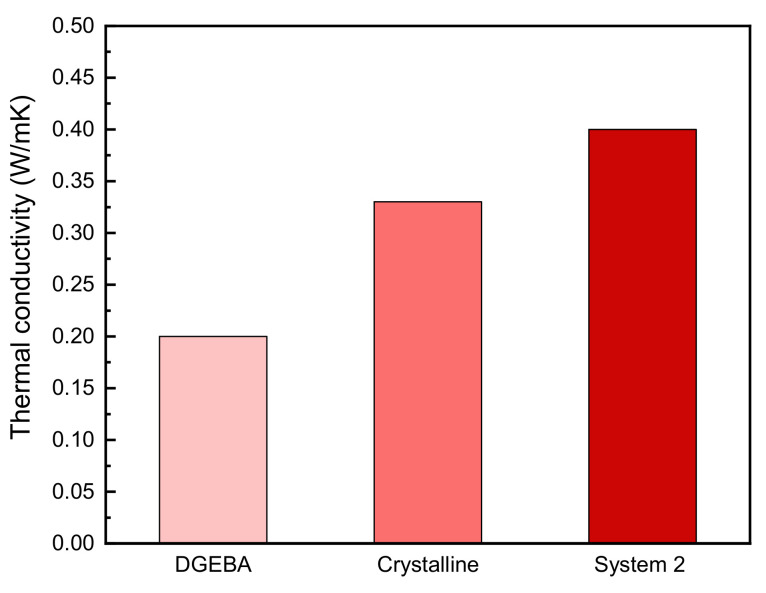
Thermal conductivities of DGEBA, crystalline epoxy resin, and System 2.

Figure 10 shows that the phase transition temperature can be modulated by using different DGP30n ratios, and it is shown that the liquid crystal form appeared in a very wide temperature range in the second system. Therefore, in systems suitable for maintaining the liquid phase during heat curing, the wide temperature range of liquid crystal is important for finding the optimal curing conditions, selecting the optimal curing agent, and investigating the curing temperature [32]. Figure 11 shows the thermal conductivity of DGEBA, crystalline epoxy resin, and System 2 that DGP304 and DGP308 is mixed in 1:1 mol ratio. The liquid-crystalline epoxy developed in this study was found to exhibit higher thermal conductivity than conventional non-liquid epoxy resin. Table 1 summarizes the thermal conductivities of each epoxy matrix with *p*-phenylenediamine (PDA).

## 4. Thermally Conductive Composites Using Liquid Crystalline Epoxies

**Figure 12:**
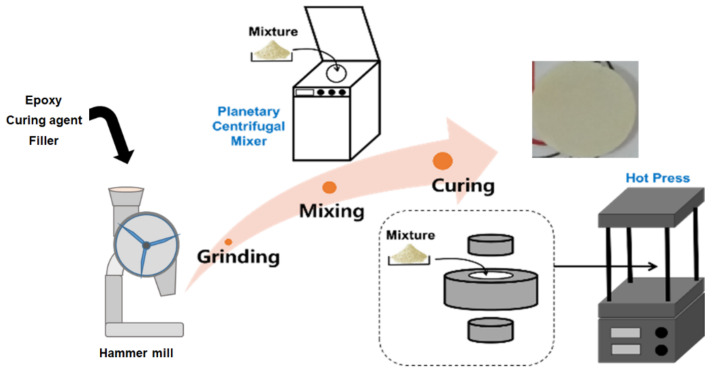
Manufacturing process for thermally conductive composites using liquid crystalline epoxies.

Since LCE exhibits a solid state at room temperature, the manufacturing process of a thermally conductive composite includes a powder mixing process employing a high-heat-dissipation ceramic powder (see Figure 12). After mixing the powder, the mixture was put into a mold, and curing was carried out under a specific heat and pressure.

**Figure 13:**
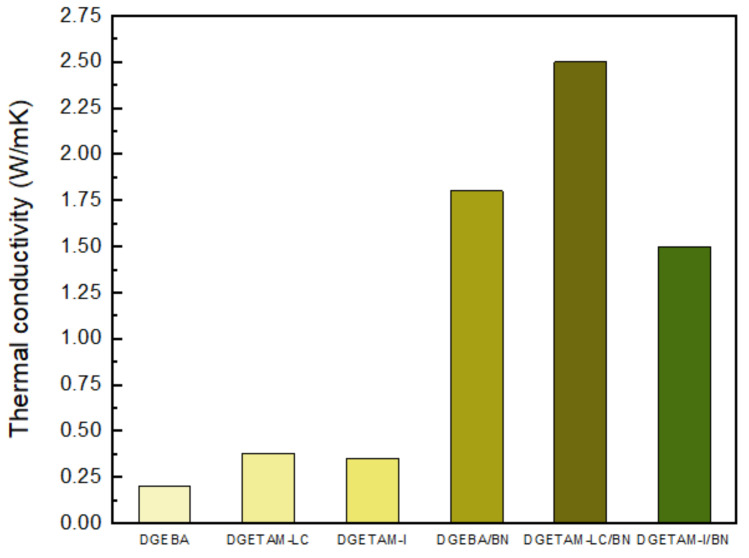
Thermal conductivities of epoxy/m-PDA and epoxy/m-PDA/BN filler 35 vol%.

The production of a high-heat-dissipation composite material using an LCE resin and hexagonal boron nitride (BN) is a good example [21]. The BN used in the complex is a highly thermally conductive filler with a platelet shape and a thermal conductivity of 60 W/mK. Filler content amounting to 0–35 vol% was added to compare the thermal conductivity of the cured material in the isotropic and liquid phases of the DGEBA epoxy and the DGEBA epoxy mixed with DGETAM (see the molecular structure in Figure 8) under the same conditions. The thermal conductivity of all the epoxy samples tended to increase with the addition of BN. The thermal conductivity in the liquid phase of the DGETAM/liquid mixture was 0.38 W/mK, which increased to 2.5 W/m K at 35 vol% filler content. Curing of the isotropic phase increased from 0.35 to 1.5 W/m K at 35 vol%. In addition, DGEBA epoxy also showed an increase in thermal conductivity to 1.8 W/m K as the BN content increased to 35 vol%. The thermal conductivities of the cured material in the liquid and isotropic phases show that the thermal conductivity of the cured material in the liquid phase is greater than that in the anisotropic phase when increasing BN content. The aggregation of *h*-BN with an anisotropically oriented liquid crystal structure in the liquid phase shows a higher increase in thermal conductivity than in the isotropic phase [21]. Figure 13 and Table 2 summarize the thermal conductivities of Epoxy/m-PDA and Epoxy/m-PDA/BN filler at 35 vol%.

Prior studies have shown that the use of LCE results in higher thermal conductivity than that obtained when using typical EPs, and when a complex is manufactured by adding a high-thermal-conductivity filler, the overall thermal conductivity becomes higher than that of the cured epoxy in the DGEBA system.

## 5. Orientation Effect on Thermal Conductivity of Liquid Crystalline Epoxies

**Figure 14:**
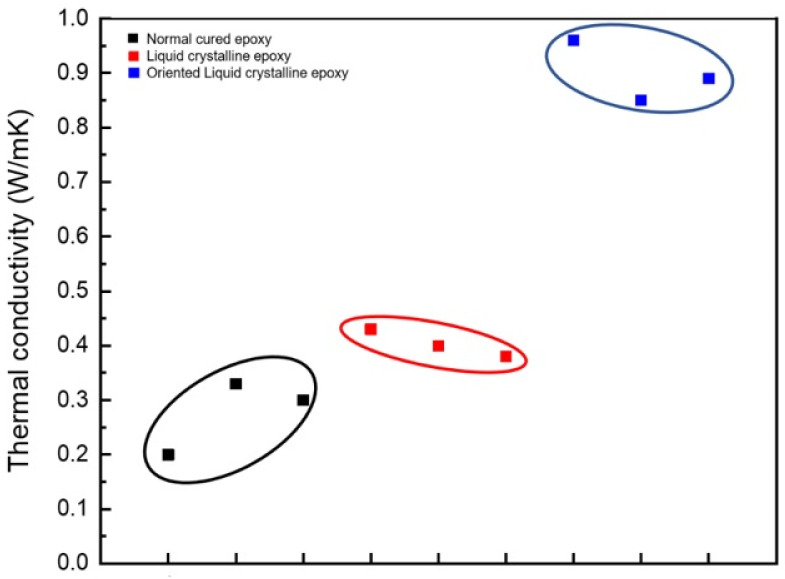
Distribution of thermal conductivity with 10 types of cured epoxy compounds.

In Figure 1, the relationship between the alignment of the liquid crystal and the improvement in the thermal conductivity is explained. In the curing process of the LCE, a magnetic field was applied to evaluate the thermal conductivity of the oriented LCE. Miyouki et al. synthesized an LCE with a thermal conductivity of 0.38 W/mK when cured with m-PDA [21]. Furthermore, the liquid crystal epoxy diglycidyl ether of terephthalyidene (DGETAM) was mixed with the curing agent, 4,4′-diaminodiphenylethane (DDE), directed along the magnetic field, and then, the cured epoxy was manufactured [33]. Figure 14 shows the structures of DGETAM and DDE. Since LCE exhibits a nematic phase at 169–202 °C, a magnetic field of 0–10 T was applied after mixing with a curing agent in this temperature range to cure at 170 °C for 15 min. The heat-diffusion coefficient was measured using the laser flash technique.

When the DGETAM/DDE was cured without applying a magnetic field, the thermal conductivity was 0.43 W/mK. However, when a 10 T magnetic field was applied in parallel with the direction of the liquid crystal’s orientation, the thermal conductivity almost doubled to a value of 0.89 W/mK. However, if the applied magnetic field was vertical to the orientation of the liquid crystal, a lower thermal conductivity of 0.35 W/mK was observed, as opposed to when the magnetic field did not exist.

The thermal conductivity when the LCE is directed along the magnetic field and cured is much higher than the thermal conductivity of the general bisphenol A epoxy (0.2 W/mK). In fact, the thermal conductivity of a polymer is affected by phonon scattering. When the polymer is cured, the magnetic field is strongly oriented and has a better aligned structure, making it easier to transmit phonons in the aligned direction and minimizing phonon scattering. The relationship between the alignment of epoxy and thermal conductivity according to the strength of the magnetic fields was also studied, which indicated that as the alignment of the liquid crystalline epoxy improves, the thermal conductivity increases accordingly.

However, above a certain magnetic field strength, the alignment of the liquid crystal epoxy does not increase. The orientation principle of a liquid crystal compound in a magnetic field is derived from the magnetic susceptibility (χ) of the benzene rings in the mesogen group in the vertical and horizontal directions of the magnetic field. Therefore, the number of benzene molecules in the liquid crystal compound and the orientation of benzene change the strength of the magnetic field required to orient the liquid crystal. In the case of compounds on a normal nematic liquid crystal, it has been reported that they can be oriented by magnetic fields greater than 1 T. It can be confirmed that a higher thermal conductivity is obtained through orientation using magnetic fields for liquid crystal compounds [18,34].

## 6. Conclusions

Recently, the thermal conductivity of polymers has become important owing to the popularity of miniaturization of electronic devices. Therefore, LCE is considered to be a polymer with high thermal conductivity. Since general EP forms a three-dimensional network structure after curing, resulting in phonon scattering, liquid epoxy can be arranged using anisotropy and cured to obtain higher thermal conductivity than general epoxy. In addition, if a strong magnetic field is applied to the liquid crystal in the aligned direction, it can have a strong network structure oriented in one direction and a high thermal conductivity [21]. It can be seen that LCE complexes have attracted attention and have been studied for future high-performance heat dissipation materials. Furthermore, research on the fabrication of highly heat-dissipating compounds using liquid-crystalline epoxy and highly thermally conductive fillers such as carbon nanotubes (CNTs) [35], alumina [36], BN [21,37], graphite [38], and graphene [39] is usually conducted.

If various types of LCEs are synthesized under magnetic fields and mixed with conductors having higher heat conductivity, such as BN and alumina, we expect the development of polymer composites with higher thermal conductivities in the future.

In the future, the heat dissipation problem is expected to emerge as a core problem that must be solved in electronic devices that are becoming smaller, thinner, and lighter. In terms of solving these problems, the liquid crystal epoxy resin introduced in this review is expected to provide a great solution to the design of polymer composite materials in terms of weight reduction and effective heat dissipation.

## Figures and Tables

**Table 1:** Thermal conductivities of DGEBA, crystalline epoxy resin, and System 2 cured with *p*-phenylenediamine (PDA). Reproduced from Ref. [31] with permission from The Royal Society of Chemistry.

Epoxy Matrix	Curing Agent	Thermal Conductivity (W/mK)
DGEBA	PDA	0.20
Crystalline	PDA	0.33
System 2	PDA	0.40

**Table 2:** Thermal conductivity of epoxy/m-PDA and epoxy/m-PDA/BN filler at 35 vol%.

Epoxy/m-PDA	Thermal Conductivity(W/mK)
DGEBA	0.20
DGETAM(LC)	0.38
DGETAM(I)	0.35
DGEBA/BN 35 vol%	1.8
DGETAM(LC)/BN 35 vol%	2.5
DGETAM(I)/BN 35 vol%	1.5
